# Human Embryonic Stem Cells

**DOI:** 10.1038/sj.bjc.6601577

**Published:** 2004-01-20

**Authors:** M Clements

**Affiliations:** 1 Wellcome Trust Research Fellow, University College London, UK

The publicity surrounding the first successful derivation of a human embryonic stem (hES) cell line in 1998 raised the expectation that stem cells will be the panacea for all diseases as well as provoking an ethical debate with respect to the way in which human embryos are used in research. ‘Human Embryonic Stem Cells’ is the first book solely dedicated to this field, covering all aspects of hES biology, providing a balanced discussion of ethical issues, as well as a detailed description of the progress that has been made in understanding how these cells function.

The book is arranged as a compilation of chapters, written by experts in the field. The first three chapters deal with both the ethical and policy issues, while the remaining 15 chapters describe methods for HES cell isolation, their propagation and differentiation, and the potential therapeutic application of this research. There is enough breadth to be informative both to the novice as well as established researchers in the field.

Human embryonic stem cells can only be isolated from the blastocyst stage embryo and hence is an ethically contentious issue. The first chapter of the book describes both sides of the ethical debate, which is mainly centred on defining the point at which life begins during development and balancing this against the potential benefits that using surplus embryos could provide to society. It explains the position of different religions and describes the national policies that various countries have implemented to regulate this research. The book also discusses the alternative sources of stem cells (both pluripotent and adult cells), explaining in this context the characteristics that make hES cells so unique. A chapter is also dedicated to the controversial subject of therapeutic cloning and provides a very balanced discussion of the ethical and technical issues involved. Overall, the sections of the book dealing with ethical issues are very well written and balanced, leaving the reader to draw their own conclusions about the morality of hES cell research.

For researchers in the US, there is a particularly useful chapter written as a researcher's guide to federally funded cell research in the US. In August 2001, President Bush banned the derivation of new human ES lines using public funds and restricted research to existing hES line. This chapter explains succinctly what research is allowed and prohibited with public funds, and also has an important section explaining the restrictions that also apply to research outside the US when performed in collaboration with US researchers. A surprising point raised in this chapter is the fact that embryo research performed with private money in the US is basically unregulated. The book also has a section on patents that infringe on hES cell research and has a great introduction to patent law for the uninitiated. This is particularly relevant for researchers who are interested in commercially exploiting hES cell technologies and explains how restrictive the original US patent is to the development of hES-based therapies. The stance of the European Patent office is also discussed and how its regulation is much more flexible, opening up greater competition than is possible in the US.

Only one chapter of the book is dedicated to detailed protocols describing the methods for deriving hES cells and the complex methods required for maintaining the cells in an undifferentiated state. It is especially useful since the original publications describing hES derivation contain very little methodological detail. This is the only chapter of the book containing detailed protocols and so anyone purchasing this book with the expectation that it will contain detailed protocols covering all aspects of hES research will be disappointed. Nevertheless, the book does contain five very comprehensive chapters describing the approaches used by different groups to differentiate hES cells to specific cell types. These chapters are up to date and very well referenced, allowing the reader to access easily the original research articles for the methodological detail. The authors draw useful comparisons between the signalling events that are known to occur *in vivo* during early embryonic development and the attempts to recreate these conditions *in vitro*. They also highlight the different responses of mouse and human ES cells to similar differentiation stimuli. When reading the book, one is struck by how much this field is in its infancy, as demonstrated by the fact that many of the chapters are based on only one or two publications describing the initial attempts to differentiate human ES cells. This is due in part to the limited number of labs that have had access to hES cell lines since their isolation in 1998. This situation is rapidly changing as the cell lines become more widely distributed around the world.

The last section of the book deals with the potential therapeutic development of hES cells. The clinical use of hES cells is still many years away, particularly since we are yet to understand the mechanisms that control the differentiation process. The authors highlight the potential barriers to using hES cells for treating a variety of diseases and discuss the various approaches being used to resolve these problems. Such barriers include immune rejection, uncontrolled proliferation and poor long-term survival of graft tissue. This section of the book gives a clear outline of the FDA regulations relevant to the development hES cell-based therapies and presents a case study of a first clinical trails using human neurone-like cell line derived from a human embryonic carcinoma line for the treatment of stroke and spinal cord injury.

In summary, this is a very comprehensive text on all aspects of hES cell biology. The book covers all of the latest developments in the field, and is very timely due to the explosion of interested in hES cells. As a consequence of the way the book has been compiled, many topics are repeated throughout the text, but since each chapter has been written by a different author, one gets many different perspectives. As access to human ES cell lines becomes more wide spread, this field will rapidly move forward and in a very short span of time this book will become outdated. However, this still remains the most comprehensive review of all aspects of the hES cell biology, and is a must for anyone planning to break into the field.

